# Generation of two iPSC lines from patients with Aicardi-Goutières syndrome carrying either biallelic *ADAR1* mutations (PC138) or a heterozygous *IFIH1* mutation (PC139)

**DOI:** 10.1016/j.scr.2025.103873

**Published:** 2025-11-27

**Authors:** Lisa Zerad, Céline Banal, Brigitte Onteniente, Nathalie Lefort, Alice Lepelley, Luis Seabra, Marie Hully, Christiane Zweier, Nadège Bondurand, Yanick J Crow, Marie-Louise Frémond

**Affiliations:** 1 Université Paris Cité, Imagine Institute, Laboratory of Neurogenetics and Neuroinflammation, F-75015 Paris, France; 2 Université Paris Cité, Imagine Institute, Laboratory of Embryology and Genetics of Human Malformation, F-75015 Paris, France; 3 Université Paris Cité, Imagine Institute, iPSC Core Facility, INSERM UMR U1163, F-75015 Paris, France; 4 Phenocell SAS, 45 Boulevard Marcel Pagnol, 06130 Grasse, France; 5 Paediatric Neurology Department, AP-HP, Necker Hospital, F-75015 Paris, France; 6 Department of Human Genetics, Inselspital Bern, University of Bern, Bern, Switzerland; 7 Institute of Human Genetics, Friedrich-Alexander-Universität Erlangen-Nürnberg, Erlangen, Germany; 8 MRC Human Genetics Unit, Institute of Genetics and Cancer, University of Edinburgh, Edinburgh, UK; 9 Paediatric Haematology-Immunology and Rheumatology Unit, AP-HP, Necker Hospital, F-75015, Paris, France

## Abstract

Mutations in *ADAR1* (Adenosine deaminase acting on RNA 1) and *IFIH1* (Interferon Induced With Helicase C Domain 1) are associated with Aicardi-Goutières syndrome (AGS), a genetically determined inflammatory disorder particularly affecting the brain and skin. Here, we generated induced pluripotent stem cells (iPSCs) from one patient carrying compound heterozygous loss-of-function mutations in *ADAR1* (PC138/AGS0788.1: c.577C>G p.(Pro193Ala) and c.1386_1390del p.(Asp462Glufs*2)), and one individual carrying a heterozygous gain-of-function mutation in *IFIH1* (PC139/AGS2177.1: c.2336G>A p.(Arg779His)). Cells from these patients were reprogrammed by episomal transfection, had normal karyotype, expressed pluripotency markers and were able to differentiate into the three germ cell layers.

## Resource Table

**Table:** 

Unique stem cell lines identifier	PCIi034-A -PCIi035-A
Alternative name(s) of stem cell lines	PCIi034-A (for PC138): AGS0788.1 PCIi035-A (for PC139): AGS2177.1
Institution	Phenocell SAS (45 Boulevard Marcel Pagnol 06130 Grasse, France)
Contact information of distributor	Yanick Crow (yanickcrow@mac.com), Marie-Louise Frémond (marie-louise.fremond@institutimagine.org)
Type of cell lines	iPSC
Origin	Human
Additional origin info required	PC138: Age: 11 years Sex: Female Ethnicity if known: Caucasian PC139: Age: 35 years Sex: Male
Cell Source	PBMC
Clonality	Clonal
Method of reprogramming	Episomal
Genetic Modification	No
Type of Genetic Modification	N/A
Evidence of the reprogramming transgene loss (including genomic copy if applicable)	PCR
Associated disease	Aicardi-Goutières syndrome (AGS)
Gene/locus	PC138: *ADAR1* c.577C >G (p.(Pro193Ala)); c.1386_1390del (p.(Asp462Glufs*2)) PC139: *IFIH1* c.2336G>A (p.(Arg779His))
Date archived/stock date	March 27, 2018
Cell line repository/bank	hPSCreg (https://hpscreg.eu/cell-line/PCIi034-A; https://hpscreg.eu/cell-line/PCIi035-A)
Ethical approval	Written informed consent to participate in biomedical research was obtained from the legal representatives of the patients. The generation, use and storage of human iPSCs were performed with approval from the Comité de Protection des Personnes (ID-RCB/EUDRACT: 2014-A01017-40, revalidated in 2022)

## Resource utility

These *ADAR1* and *IFIH1*-mutated-iPSCs, derived from two patients with Aicardi-Goutières syndrome (AGS), will allow the development of iPSC-derived models with which to explore the cellular mechanisms leading to disease, and to evaluate potential novel therapies.

## Resource Details

Mutations in human *ADAR1* and *IFIH1* cause Aicardi-Goutières syndrome (AGS), a genetically determined inflammatory disorder particularly affecting the brain and skin (Rice and al, 2012; Rice et al, 2014), belonging to a broader disease grouping referred to as the type I interferonopathies (Stetson and Crow, 2022). These diseases are characterized by chronic activation of the type I interferon signaling pathway. Patients with AGS demonstrate heterogeneous phenotypes including intracranial calcification, white matter abnormalities and cerebral atrophy. Peripheral neuropathy has been described in some patients, suggesting a role of ADAR1 in the peripheral nervous system including neural crest cell development (Crow et al, 2015).

**Fig 1:**
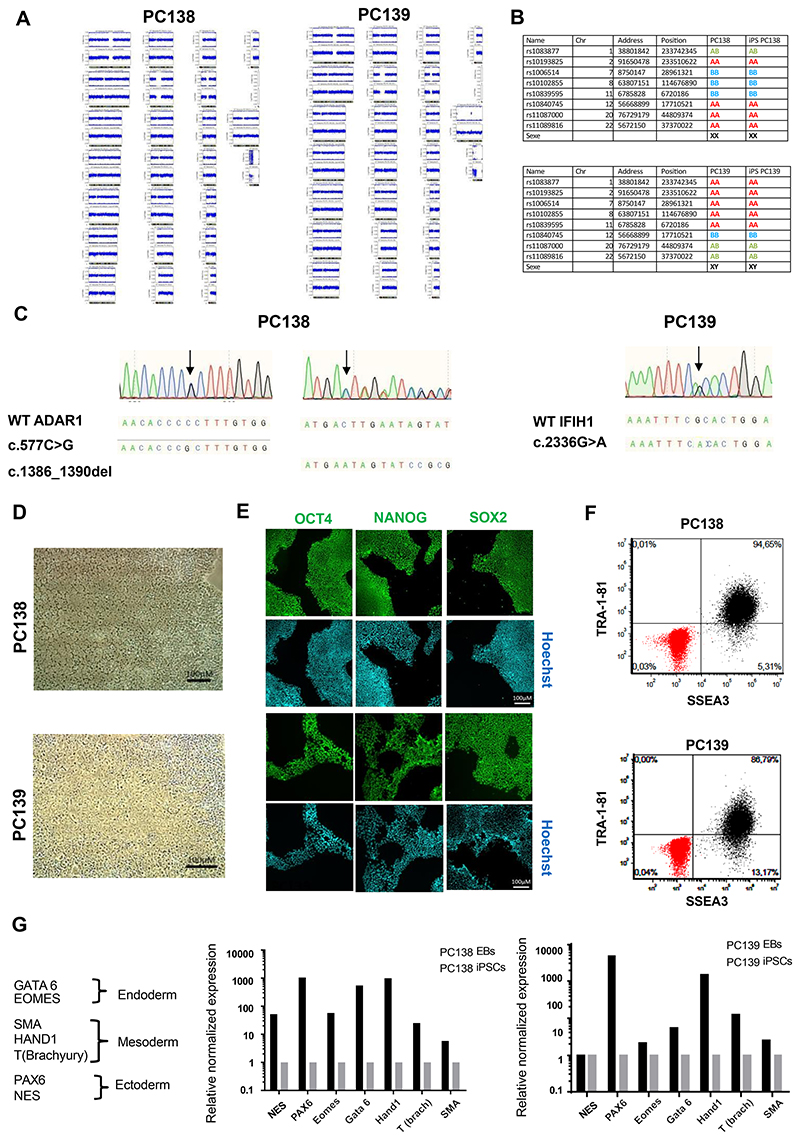


In this study, we report the generation and characterization of iPSC lines derived from cryopreserved peripheral blood mononuclear cells (PBMCs) from an 11-year-old female patient compound heterozygous for two loss-of-function mutations in *ADAR1* (c.577C>G; p.(Pro193Ala) and c.1386_1390del; p.(Asp462Glufs*2)), and a 35-year-old male patient carrying a gain-of-function pathogenic mutation in *IFIH1* (c.2336G>A; p.(Arg779His)). Patient PBMCs were reprogrammed into iPSCs using episomal vectors. Colonies with a morphology typical of pluripotent stem cells were individually and manually selected to establish a feeder-free iPSC line. To determine the quality of the generated iPSCs, several genotypic and functional assays were performed (Table 1). Karyotyping showed normal chromosome numbers and structures in both cell lines (Supplementary Figure 1A). Analysis of genomic integrity was performed using a comparative SNP analysis of parental PBMCs with the iPSC line (Fig. 1A). SNP arrays have a high enough resolution to allow the detection of sub-chromosomal defects. This technique can also detect copy-neutral loss of heterozygosity events, but does not allow for the detection of balanced rearrangements or low-level mosaicism. Thus, to confirm the absence of aneuploidies, deletions or insertions, we performed a genome SNP array analysis of iPSCs and parental PBMCs. The origin of the iPSCs was confirmed by matching genotypes with starting PBMCs (selected SNPs in Fig.1B). Sequencing confirmed the presence of the expected mutations in the relevant iPSC lines (*ADAR1*: PC138; *IFIH1*: PC139) (Fig. 1C, black arrows). The generated cell lines displayed morphological features consistent with iPSC-identity assessed by phase contrast microscopy (Fig. 1D). Furthermore, we assessed the expression of pluripotent stem cell markers through immunocytochemical staining of OCT4, NANOG and SOX2 (Fig. 1E), and by flow cytometry of TRA-1-81 and SSEA-3 (Fig. 1F). The ability to differentiate spontaneously into cells of the three germ layers was confirmed by embryoid body (EB) formation and trilineage differentiation potential through quantitative real-time PCR (qRT-PCR). Both the PC138 and PC139 cell lines efficiently differentiated into endodermal (*GATA6* and *EOMES*), mesodermal (*HAND1, T(brach)* and *SMA*) and ectodermal (*PAX6*) lineages, expressing the appropriate specific layer markers (Fig. 1G). The two cell-lines were free of any mycoplasma contamination (Supplementary Figure 1B).

## Materials and Methods

### Generation of iPSCs

Patient PBMCs were purified using a Ficoll-Paque layer and 1.5x10^6^ cells reprogrammed with episomes (Epi5™ Reprogramming Kit, ThermoFisher #A15960) using Amaxa nucleofector program T-016. Transfected cells were seeded on laminin-521-coated tissue culture plates (Falcon) and incubated with StemSpan™ SFEM II for 7 days. Medium was progressively switched to StemMACS™ (Miltenyi Biotec) over 10 days, then changed daily. Manually picked small colonies were transferred to Geltrex™ matrix-coated dishes (ThermoFisher) for development. iPSCs were maintained in mTeSR1 culture medium (StemCell Technologies) in hESC-qualified Matrigel (ThermoFisher) coated wells, and passaged with StemPro Accutase (Thermo Fisher) every week for 10 weeks. All cells were grown at 37°C, in 5% CO2 in a humidified incubator.

### In vitro trilineage differentiation by embryoid body (EB) formation

iPSCs (passage 10) were harvested with Accutase, centrifuged at 290g/3min, and then plated at 10^4^ cells per well in a 96-well Ultra-Low Attachment plate (Costar #7007). Cells were cultured in EB medium (Merck #SCM018) supplemented with 10mM Blebbistatin (Selleckchem S7099). After one week, EBs were transferred in Eppendorf microtubes for RNA extraction and qRT-PCR (*GATA6, EOMES* (endoderm); *HAND1, T(brach), SMA* (mesoderm); *PAX6* (*ectoderm))*.

### qRT-PCR

RNA extracted from iPSCs (passage 10) using the Isolate II RNA mini kit (Bioline) on 350 µL of cell lysate was quantified on a Varioskan™ Lux microplate reader at 260nm. Reverse transcription was performed on 250ng RNA using the SensiFast Synthesis Kit (Bioline), and qPCR conducted with iTAQ™ SYBR Green Supermix (Biorad) using the CF96 Real-Time PCR detection system (Biorad). Results were normalized against *18S/CREBBP*.

### Immunofluorescence

iPSCs (passage 10) were fixed for 12min with 4% paraformaldehyde, permeabilized with 0.4% Tween20 and blocked with 10% FBS at RT. Primary and secondary antibodies (Table 2) were incubated in 4% FBS/0.1% Tween, overnight/4°C and 1h/RT, respectively. Nuclei were stained with 1µg/ml DAPI (Promega). The expression levels of OCT4, NANOG, and SOX2 were quantified on a CellInsight™ CX5 epifluorescent microscope (ThermoFisher).

### DNA isolation, sequencing and karyotype analysis

DNA was isolated from iPSCs (passage 10) using the PureLink™ Genomic DNA Mini Kit (Invitrogen). Sanger sequencing confirmed the presence of the compound heterozygous c.577C>G and c.1386_1390del *ADAR1* mutations, and the heterozygous c.2336G>A *IFIH1* mutation (primers in Table 2). Karyotypic analysis was performed at the Chromostem platform (Montpellier CHU, France) on 10 (PC138) and 11 (PC139) metaphasic cells (passage 10) employing the RHG banding method (400 band resolution). Cells were incubated with Accutase at 37°C for 5min, washed and centrifuged, and then subjected to hypotonic swelling and fixation before analysis. Molecular karyotyping was performed using the Infinium Core-24 v1.2 Kit (Illumina), containing 300,000 SNPs. Data were analysed with BeadStudio/GenomeStudio software (Illumina).

### Flow cytometry

iPSCs (passage 10) were harvested with Accutase™ (StemCell Technologies #07920) and centrifuged at 300g/3min. Antibodies (Table 2) were added to cells resuspended in Live Cell imaging solution (Life Technologies #A14291DJ). Mixtures were incubated for 30min/RT in the dark, and centrifuged at 300g/3min. Cell pellets were resuspended in 200µL Live Cell imaging solution, and analysed using the Accuri™ C6 Plus flow cytometer (BD Biosciences).

### Mycoplasma detection

Mycoplasma testing of iPSCs (passage 5 and 10) was performed using the MycoAlert™ Mycoplasma Detection Kit (Lonza).

### STR analysis

The STR analysis for the two cell lines (Fig 1A and B) has been uploaded (file named ‘STR analysis’), but are not for publication so as to protect donor identity.

## Supplementary Material

Supplementary

## Figures and Tables

**Table 1:** Characterization and validation

Classification	Test	Result	Data
**Morphology**	Phase contrast bright field microscopy	Normal morphology	Fig 1, panel D
**Phenotype**	Qualitative analysis by immunocytochemistry	Expression of the pluripotency markers: OCT4, SOX2 and NANOG	Fig 1, panel E
	Quantitative analysis by Flow cytometry	PC138 : SSEA3+TRA-1-81: 94.65 % PC139 : SSEA3+TRA-1-81: 86.79%	Fig 1, panel F
**Genotype**	Karyotype (G-banding) and resolution	Resolution 400-500 bands PC138: Normal, female 46XX PC139: Normal, male 46 XY	Supplementary Figure 1A
**Identity**	Microsatellite PCR (mPCR) OR STR Analysis		STR analysis
**Mutation analysis (IF APPLICABLE)**	Sequencing	PC138: compound heterozygous mutations in *ADAR1* (c.577 C>G; c.1386_1390del) PC139: heterozygous mutation in *IFIH1* (c.2336 G>A)	Fig 1, panel C
	Southern blot OR WGS	N/A	N/A
**Microbiology and virology**	Mycoplasma	Mycoplasma testing by luminescence. Negative	Supplementary Figure 1B
**Differentiation potential**	Embryoid body formation	qRT-PCR of specific layer markers: - Endoderm: GATA6,EOMES - Ectoderm: PAX6 - Mesoderm: SMA, HAND1	Fig 1, panel G
**List of recommended germ layer markers**	Expression of these markers has to be demonstrated at mRNA (RT PCR) or protein (IF) levels, at least 2 markers need to be shown per germ layer	qRT-PCR of specific layer markers: - Endoderm: GATA6,EOMES - Ectoderm: PAX6 - Mesoderm: SMA, HAND1	Fig 1, panel G
**Donor screening (OPTIONAL)**	HIV 1 + 2 Hepatitis B, Hepatitis C	Negative	not shown
**Genotype additional info (OPTIONAL)**	Blood group genotyping	N/A	N/A
	HLA tissue typing	N/A	N/A

**Table 2:** Reagents details

	Antibodies used for immunocytochemistry/flow-cytometry			
	Antibody	Dilution	Company Cat #	RRID
**Pluripotency markers**	Anti-OCT4	1:500	Millipore Cat#ab18976, RRID: AB_444714	
	Anti-SOX2	1:1000	Millipore Cat#AB5603, RRID: AB_2286686	
	Anti-NANOG	1:1000	Millipore Cat#ab9220, RRID: AB_11213156	
	PE anti human SSEA-3	1:500	Biolegend Cat#330406, RRID: AB_1089206	
	TRA-1-81-APC	1:500	Biolegend Cat# 330702, RRID: AB_1089240	
**Secondary antibodies**	AlexaFluor 647 Goat anti-Rabbit IgG (H + L)	1/1000	Invitrogen Cat#A-21244, RRID: AB_2535812-	
	AlexaFluor 488 Goat anti-Mouse IgG (H + L)	1/1000	Invitrogen Cat#A-11001, RRID: AB_2534069	
	**Primers**			
	**Target**	**Size of band**	**Forward/Reverse primer (5′-3′)**	
**Episomal plasmid (qPCR)**	ORiP from pCEP4		F : TTCCACGAGGGTAGTGAACC R : TCGGGGGTGTTAGAGACAAC	
	ORiP from pCEP4		F : ATCGTCAAAGCTGCACACAG R : CCCAGGAGT(CCAGTAGTCA	
	EBNA from episomal vector		F : ATCAGGGCCAAGACATAGAGATG R : GCCAATGCAACTTGGACGTT	
**Differentiation markers (qPCR)**	CREBBP		F: GAGAGCAAGCAAACGGAGAG R: AAGGGAGGCAACAGGACA	
	18S		F: GAGGATGAGGTGGAACGTGT R: TCTTCAGTCGTCCAGGTCT	
	PAX6		F: AGTGAATCAGCTCGGTGGTGTCTT R: TGCAGAATTCGGGAAATGTCGCAC	
	NES (nestin)		F : GCGTTGGAACAGAGGTTGGA R : TGGGAGCAAAGATCCAAGAC	
	EOMES		F: AAGGCTTCAGAGACAACTATG R: CGACCTCCAGGGACAATC	
	GATA 6		F: AAAGACTTGCTCTGGTAATAGC R: GGCTGTAGGTTGTGTTGTGG	
	HAND1		F: TCAAGGCTGAACTCAAGAAGG R: CGGCTCACTGGTTTAACTCC	
	Housekeeping gene (CREBBP)		F: GAGAGCAAGCAAACGGAGAG R: GAGAGCAAGCAAACGGAGAG	
	SMA		F: GACCGAATGCAGAAGGAGAT R: CACCGATCCAGACAGAGTATT	
	T (brachyury)		F: TAGCGAGAAATATGCCGAGGAG R: GAGCTGCGTGATCCGATGG	
**Targeted mutation analysis/sequencing**	ADAR1 (p.(Pro193Ala))	500bp	F: CACCTTCCCTCCCAGGACT R: GTCTTCCGGTTCCAAACTCG	
	ADAR1 (p.(Asp462Glufs*2))	511bp	F: CTCCTCTGCCCTGAATTTGG R: GAAGGAGGGCATCTCCATGA	
	IFIH1 (p.(Arg779his))	266bp	F: GATTTTAATGTGTTTAGCATCACAAA R: GCAATTAAAATAGGAACACAACAAA	
