# Supplementary material for: Generation of two iPSC lines from patients with Aicardi-Goutières syndrome carrying either biallelic ADAR1 mutations (PC138) or a heterozygous IFIH1 mutation (PC139)
Source: Stem Cell Res. Author manuscript; Available in PMC 2025 Dec 29. (PMC7618548; doi:10.1016/j.scr.2025.103873)
Supplement: Supplementary [file EMS211642-supplement-Supplementary.docx]

**Supplementary Figure 1: Karyotyping and mycoplasma testing of PC138 and PC139.**

**A**


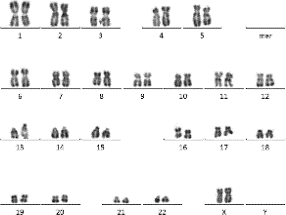

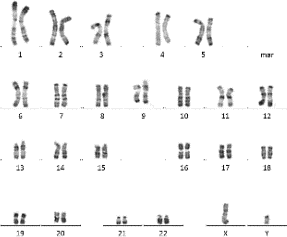


**PC138 PC139**

**B**

**Mycoplasma test before thawing of PC138 and PC139**

|  | **Positive control** | **Negative control** | **PC138 (AGS0788.1)** | **PC139 (AGS2177.1)** |
| --- | --- | --- | --- | --- |
| **A** | 168 | 260 | 144 | 108 |
| **B** | 5168 | 40 | 32 | 48 |
| **B/A** | 30.76 | 0.15 | 0.22 | 0.44 |
|  | Positive | Negative | Negative | Negative |

**Mycoplasma test after PC138 and PC139 sample banking (passage 5)**

|  | **Positive control** | **Negative control** | **PC138 (AGS0788.1)** | **PC139 (AGS2177.1)** |
| --- | --- | --- | --- | --- |
| **A** | 192 | 264 | 68 | 86 |
| **B** | 7968 | 60 | 36 | 18 |
| **B/A** | 41.500 | 0.227 | 0.53 | 0.21 |
|  | Positive | Negative | Negative | Negative |

**Mycoplasma test after PC138 and PC139 sample banking (passage 10)**

|  | **Positive control** | **Negative control** | **PC138 (AGS0788.1)** | **PC139 (AGS2177.1)** |
| --- | --- | --- | --- | --- |
| **A** | 160 | 232 | 196 | 128 |
| **B** | 3048 | 52 | 160 | 104 |
| **B/A** | 19.05 | 0.22 | 0.82 | 0.81 |
|  | Positive | Negative | Negative | Negative |

Mycoplasma testing by luminescence was negative.
